# Supplementary material for: Genome-Wide Identification and Expression Analyses of Odorant-Binding Proteins in Hoverfly Eupeodes corollae
Source: Int J Mol Sci. 2025 Sep 14;26(18):8956. doi: 10.3390/ijms26188956 (PMC12469980; doi:10.3390/ijms26188956)
Supplement: Supplementary file 1 [file ijms-26-08956-s001.zip › Tables S1-S3.pdf]

Article

Genome-Wide Identification and Expression Analyses of Odorant-Binding Proteins in Hoverfly *Eupeodes corollae*

He Yuan [1,†], Huiru Jia [2,†], Xianyong Zhou [2], Hui Li [1], Chao Wu [3] and Kongming Wu [1,*]

1. State Key Laboratory for Biology of Plant Diseases and Insect Pests, Institute of Plant Protection, Chinese Academy of Agricultural Sciences, Beijing 100193, China; yuanhe1001@126.com (H.Y.); lihuilh521@163.com (H.L.)
2. Xianghu Lab, Hangzhou 311258, China; jhuiru@163.com (H.J.); ZhouXY160721@163.com (X.Z.)
3. Shenzhen Branch, Guangdong Laboratory of Lingnan Modern Agriculture, Genome Analysis Laboratory of the Ministry of Agriculture and Rural Affairs, Agricultural Genomics Institute at Shenzhen, Chinese Academy of Agricultural Sciences, Shenzhen 518000, China; wuchao@caas.cn

* Correspondence: wukongming@caas.cn

† These authors contributed equally to this work.

Table S1 Primers used in this study

Gene	Forward primers (5'-3')	Reverse primers (5'-3')
EcorOBP5	ATGAAACACTTCGTACTTGTGTT	TTATTTCAATTCACTTCTAATGCA
EcorOBP14	ATGAAATATTTGTGTGTTGTTTTTG	TTTCAATTCACTAGAAATACACATG
EcorOBP19	TTTAGTCACCTTCGTTTTGTTATTG	CATTTCTTTGAAGGTCCATATATTCC
EcorOBP21	ATGAAGTTTATACTATTAGTTGCTATTATTG	TTAAAATTGTAAACTATTTTCACTTTTC
EcorOBP23	ATGAAGTTTGTACTATTATTTGCTATTATT	TTAAAATTTAAAAATATCATCACGC
EcorOBP35	ATGAAGTACACTGTAGCCCTAACG	CGAACACTTTATCAGAATGATAGTG
EcorOBP36	ATGAAGAAAATTACATTTATTGGTAAA	TCTTCATGTGATTAACTATGAATCC
EcorOBP37	ATGAATAAGTCAGTCGTCGTAAC	CTTAAAGGGTCTATCACACTCATC
EcorOBP38	ATGTCAATTCTTAACTTATTATTACTTTCTC	TTATGAATTGTTTTCAAAACCATC
EcorOBP39	ATGGATAAGAAAGTCTTCATCTTACTC	CAGTAAATAATAAATCTAACAACATAAATCC
EcorOBP40	ATGAAGCAATTCGAGTCTTCG	TTAAGGGAATTTAAAACTTGATGG
EcorOBP41	ATGGCATATTTAATAAAAGTGTTGA	TCAATAGGGAACATCGATCTT
EcorOBP42	ATGCGTACGTATTTACTTGCC	CCGGAAATGGTCTAAACAATAG
EcorOBP43	ATGGTCATTAAATTATTTGGATTC	TTGATTTATATATTTCTTATGGTAGTCATC
EcorOBP44	ATGAAGAAGATATTTTTTTCACTATTAA	TTATTCTTCTTTATCTAACTCAGGC
EcorOBP45	ATGCCTTCACTTCGGTTAAAC	TTAAGACTTTGAGTTTTTCAATTTTG
EcorOBP46	ATGGCAAACCTCAACAGAAG	TTAAAAAAACGGAGCACTTTTG
EcorOBP47	GGAGAGAGAAGTTATTGCGACG	CCATTTGATAAGCACATTCGCA
EcorOBP48	ATGCTGATGGTGCTGGAG	TTAAAAATACCGACTTATCTTATCGC
EcorOBP49	ATGTACGAATGGAATCAAATTC	CTACTCTTCTTGGATTTTCCTTTC

EcorOBP50	ATGAAAATTTTTATTATTCTTGCTCT	TTAATCCAATTTCTGCAATAACTT
EcorOBP51	ATGAAGTTCTTCTTGGTACTCACA	TTATTTTCCATTAGCGGGATT
EcorOBP52	ATGAAATTCTTTTTAACAATTATCACC	TCAATTCAGTTTACTTGTTTGTATTTC
EcorOBP53	ATGGCCAGTATAGTTGTAATTTTATT	CTATTCCTTGTTTTCTTTTTCTTTT
EcorOBP54	ATGCCAAAAGTAGCTATATTAGTATTTG	TTAATTGTTTACATCAGCATAGAATTG

Table S2 Genome assembly used in this study

Species	GenBank accession	Reference
Episyrphus balteatus	GCA_945859705.1	
Scaeva pyrastri	GCA_905146935.1	
Eristalis tenax	GCA_905231855.1	
Eupeodes corollae	GCA_042604645.1	Yuan et al. (2022)

Table S3 Homologous OBP gene pairs in *Eupeodes corollae* with other hoverflies species

	E.corollae	*Episyrphus balteatus*	*Scaeva pyrastri*	*Eristalis tenax*
1	EcorOBP1	rna-XM_055981586.1 (EbalOBP2)	Spyr025652.1	Eten010527.1
2	EcorOBP2	rna-XM_056000359.1	Spyr010234.1	Eten008166.1
3	EcorOBP4	rna-XM_056002658.1 (EbalOBP3)	Spyr024931.1	Eten010764.1
4	EcorOBP5	rna-XM_056000059.1	Spyr011225.1	Eten021705.1
5	EcorOBP7	rna-XM_055996183.1	Spyr021095.1	Eten013604.1
6	EcorOBP9	rna-XM_055994489.1 (EbalOBP13)	Spyr016449.1	——
7	EcorOBP10	rna-XM_055994002.1 (EbalOBP12)	Spyr021101.1	Eten013600.1
8	EcorOBP11	rna-XM_055994165.1 (EbalOBP14)	——	——

9	EcorOBP13	rna-XM_055994737.1	Spyr021104.1	——
10	EcorOBP14	rna-XM_056001302.1	——	——
11	EcorOBP15	rna-XM_055992316.1 (EbalOBP23)	Spyr019907.1	Eten014846.1
12	EcorOBP16	rna-XM_056000948.1	Spyr012832.1	Eten004254.1
13	EcorOBP17	rna-XM_055990230.1 (EbalOBP24)	——	——
14	EcorOBP18	rna-XM_055981474.1 (EbalOBP25)	Spyr024839.1	——
15	EcorOBP19	rna-XM_056000061.1	Spyr011199.1	——
16	EcorOBP20	rna-XM_055987247.1	——	Eten006616.1
17	EcorOBP21	rna-XM_055998043.1 (EbalOBP32)	Spyr011197.1	Eten021696.1
18	EcorOBP26	——	Spyr011194.1	——
19	EcorOBP28	rna-XM_055998044.1 (EbalOBP33)	——	——
20	EcorOBP30	rna-XM_056000434.1	Spyr011219.1	Eten021704.1
21	EcorOBP31	rna-XM_055998045.1 (EbalOBP35)	——	——
22	EcorOBP39	——	Spyr011193.1	——
23	EcorOBP40	rna-XM_056000802.1 (EbalOBP17)	Spyr009087.1	——
24	EcorOBP41	rna-XM_055989329.1	Spyr003372.1	——
25	EcorOBP42	rna-XM_055986480.1	Spyr005213.1	Eten007327.1
26	EcorOBP44	rna-XM_055999846.1 (EbalOBP19)	Spyr012831.1	Eten004253.1
27	EcorOBP45	rna-XM_055983300.1	——	——
28	EcorOBP46	rna-XM_055980505.1 (EbalOBP4)	Spyr026494.1	Eten010650.1
29	EcorOBP48	rna-XM_055994986.1	Spyr021102.1	Eten013578.1
30	EcorOBP49	rna-XM_055995592.1	——	——
31	EcorOBP50	rna-XM_055994166.1	Spyr021094.1	——
32	EcorOBP51	rna-XM_055996843.1	Spyr021091.1	——
33	EcorOBP53	rna-XM_055994521.1	Spyr017512.1	——

Note: Some of the orthologous OBPs in *E. balteatus* were named through NCBI blast.
